# Improving the Security and QoE in Mobile Devices through an Intelligent and Adaptive Continuous Authentication System

**DOI:** 10.3390/s18113769

**Published:** 2018-11-04

**Authors:** José María Jorquera Valero, Pedro Miguel Sánchez Sánchez, Lorenzo Fernández Maimó, Alberto Huertas Celdrán, Marcos Arjona Fernández, Sergio De Los Santos Vílchez, Gregorio Martínez Pérez

**Affiliations:** 1 Department of Information and Communications Engineering (DIIC), University of Murcia, 30100 Murcia, Spain; josemaria.jorquera@um.es (J.M.J.V.); pedromiguel.sanchez@um.es (P.M.S.S.); gregorio@um.es (G.M.P.); 2 Department of Computer Engineering (DITEC), University of Murcia, 30100 Murcia, Spain; lfmaimo@um.es; 3 Telecommunications Software & Systems Group, Waterford Institute of Technology, Co., X91 K0EK Waterford, Ireland; 4 Innovation and Labs, ElevenPaths, Cybersecurity Unit of Telefónica Digital España, 29071 Málaga, Spain; marcos.arjona@11paths.com (M.A.F.); ssantos@11paths.com (S.D.L.S.V.)

**Keywords:** cybersecurity, continuous authentication, adaptability, sensors, applications, mobile devices, machine learning, anomaly detection

## Abstract

Continuous authentication systems for mobile devices focus on identifying users according to their behaviour patterns when they interact with mobile devices. Among the benefits provided by these systems, we highlight the enhancement of the system security, having permanently authenticated the users; and the improvement of the users’ quality of experience, minimising the use of authentication credentials. Despite the benefits of these systems, they also have open challenges such as the authentication accuracy and the adaptability to new users’ behaviours. Continuous authentication systems should manage these challenges without forgetting critical aspects of mobile devices such as battery consumption, computational limitations and response time. With the goal of improving these previous challenges, the main contribution of this paper is the design and implementation of an intelligent and adaptive continuous authentication system for mobile devices. The proposed system enables the real-time users’ authentication by considering statistical information from applications, sensors and Machine Learning techniques based on anomaly detection. Several experiments demonstrated the accuracy, adaptability, and resources consumption of our solution. Finally, its utility is validated through the design and implementation of an online bank application as proof of concept, which allows users to perform different actions according to their authentication level.

## 1. Introduction

Continuous authentication systems for mobile devices aim to identify the owner of the device permanent and periodically but not only at a given moment, as traditional systems do. This approach provides several advantages like, for example, the improvement of both the level of security and the user’s quality of experience (QoE) during the interaction with applications requiring authentication. The fact of having the user permanently authenticated, and not from time to time, contributes to providing a higher level of security and confidence compared to traditional methods. Additionally, continuous authentication systems minimise the use of credentials during authentication processes [1]. The previous aspects justify the relevance of continuous authentication systems in the present and near future due to the amount of heterogeneous contexts with privacy concerns where sensitive or confidential information should be protected.

The lifecycle of continuous authentication systems starts by modelling the users’ behaviour when they interact with their mobile device for a given period of time (usually, some weeks is sufficient). Once the data is acquired, it is preprocessed and stored in a dataset that contains relevant information about the users’ behaviour patterns. To generate an accurate dataset with the user’s profile, the right selection of characteristics or features belonging to different dimensions of the device (sensors, applications, communications, screen gestures, etc.) is critical. Once the profile has been generated, the last step consists in the comparison of the current mobile usage with the well-known user’s behaviour stored in the dataset. This comparison is performed in real time and usually made using semi-supervised or unsupervised Machine Learning (ML) techniques [2]. At this point, it is important to highlight the suitability of using ML techniques. The set of different behaviours obtained from the device while it is operated by the user is assumed to form a manifold embedded in the space of features [3]. ML techniques try to compute an estimation of this region of the space. According to that, a new behaviour is considered an anomaly if it is far away from this area. The manifold can be so complex that it may not be easily represented by other simpler mechanisms as, for example, those based on rules.

Despite the relevant benefits provided by current continuous authentication systems, they also have open challenges that still require more effort and work from a research point of view. Among them, we highlight the following ones:

- *The selection of dimensions and features allowing for modelling the user’s behaviour in a precise and effective fashion.* The combination of several dimensions and features of mobile devices is one of the critical aspects to obtain a great accuracy during the authentication process. Currently, most of the proposals found in the literature only consider features belonging to one dimension [4]. This fact reduces the accuracy of the authentication system by obviating anomalous behaviours detectable through complementary dimensions.
- *The adaptability of the authentication systems to changes in the user’s behaviour.* The decision of how and when the user’s profile should be updated with new behaviours is critical to reach the desired adaptability of continuous authentication systems. Additionally, forgetting old behaviours is also an important aspect of an adaptable authentication system. In this context, the majority of the existing solutions do not take into account adaptability aspects when they authenticate their users [5].
- *Continuous authentication enables a complete set of attack vectors for real-time behaviour spoofing.* Innovative spoofing techniques embedded at impersonating bots allow real-time human dynamics acquisition and simulation, enabling precise behaviour replication on the user side [6]. Integrating authentication mechanisms should also involve additional recognition measures to avoid artificial actors—for instance, using non-simulable dimensions such as traceable hardware sensors.

With the goal of improving the previous challenges, the main contribution of this paper is the design and implementation of an intelligent and adaptive system of continuous authentication for mobile devices. The proposed solution relies on modelling and creating users’ profiles that contain data and features related to the usage of the applications and sensors of the device. ML-based techniques based on anomaly detection [7] are considered by our solution to measure the level of similarity between the current usage of the device and the well-known usage. Different experiments provided promising results in terms of accuracy, adaptability and resource consumption of the proposed solution. Finally, to demonstrate the utility of our intelligent continuous authentication system, we have designed and implemented an online bank application that allows users to perform different sensitive actions according to their authentication level.

The remainder of the paper is structured as follows. Section 2 discusses some related work from the academia and industry focused on continuous authentication for mobile devices. Section 3 shows the design details of the proposed solution. Section 4 shows the different decision to implement the proposed intelligent and adaptive continuous authentication system. Section 5 depicts different experiments that support the design decisions and demonstrate the suitability of our solution. Section 6 highlights the improvement of our solution with regard to the existing ones. A use case is shown in Section 7 to demonstrate the utility of our system. Finally, conclusions and future work are drawn in Section 8.

## 2. Continuous Authentication Systems

This section reviews and analyses, from different perspectives, existing continuous authentication systems belonging to the academia and industry.

### 2.1. Continuous Authentication in the Academia

Among the continuous authentication solutions that we can find in the academia, one of the most relevant is focused on identifying users through typing patterns and biometric behaviour [8]. Specifically, the authors acquired data such as rotation, vibration, or pressure by considering touchscreens and sensors of mobile devices. Once the data was collected, they used semi-supervised learning techniques to classify the profile according to the user’s behaviour. Another relevant article is presented by Patel et al. in [4]. In this article, the authors performed an interesting analysis about the different dimensions and ML algorithms that can be used in continuous authentication systems. The analysed dimensions were facial recognition, gestures, applications and location. The main conclusion of the authors was that the fact of merging data belonging to different dimensions allows for obtaining better results in terms of accuracy and error rate. On the other hand, Ehatisham-ul-Haq et al. in [9] presented a solution based on the use of sensors (accelerometer, gyroscope, and magnetometer) to identify the users’ interaction with their device. Based on the users’ habits, the authors inferred different positions where the device could be located (pocket, at waist height, in the upper arm, and on the wrist). In addition, after performing several tests with different classification algorithms such as k-Nearest Neighbours [10], Bayes Net [11], Decision Tree [12] and Support Vector Machines (SVM) [13], they concluded that the SVM algorithm is not appropriate for mobile devices due to its high computational consumption. In contrast, Bayes Net is more appropriate due to its trade-off between accuracy and consumption. Once the device position is determined, the system calculates the Euclidean distance between the evaluated instance and the same position training instances.

Another interesting work was presented by Fridman et al. in [14]. The authors considered different features like the text written by the user, location, applications usage, and visited websites. These features were evaluated separately to later combine the results obtained from the classification process and determine if the user is authorised or not. A recent work of special interest is proposed by Centeno et al. in [15]. This article proposed to perform the ML processes in the cloud, reducing the consumption load in the device and making possible the execution of complex algorithms. In this sense, they used Autoencoders to authenticate users, which allows a greater precision and resources consumption by doing the operations on the cloud. Li et al. [16] evaluated the combination of telephone calls, text messages, and application statistics to recognise users. In addition, the vectors generated on each of these dimensions included data of the location of the device to provide extra information. This study was carried out with 76 volunteers and the vectors of each of them were labelled. Finally, the authors demonstrated that the use of dynamic datasets has a positive impact on the system accuracy.

It is also worth noting the work done by Li et al. [17], where the authors proposed a system that applies data augmentation techniques [18], such as scaling, cropping, and jittering, on data obtained from accelerometer and gyroscope sensors. Data augmentation improves the generalisation capacity of the system. The ML method selected was One-Class SVM [19] after comparing it to others like Kernel Ridge Regression (KRR) [20] and k-Nearest Neighbours (kNN). Another interesting solution is the proposed by de Fuentes et al. [21]. The authors of this work used non-assisted sensors, such as battery, transmitted data, ambient light and noise to authenticate the user. This study collected information of 50 users over 24 months and then used supervised ML classification algorithms to identify the users. Specifically, they used k-NN, Adaptive Hoeffding Tree [22] and Naive Bayes [23] algorithms. Finally, SenGuard [24] is a continuous authentication system that combines movement data, voice, location, and touches on the screen. The authors of this solution proposed classification algorithms since they had information belonging to four users.

### 2.2. Continuous Authentication in the Industry

The industry also is interested in the continuous authentication topic. One of the most relevant solutions that we can find in the Android and iOS markets is the one provided by BehavioSec (San Francisco, CA, USA) [25]. Through the use of behavioural biometric techniques, features belonging to users’ behaviours are acquired and combined to guarantee a good accuracy of detection of anomalous behaviour. This company offers a software development kit (SDK) for integrating the system in client’s applications, and this SDK is available for Android, iOS and Web Browser apps. Another interesting solution is presented by Veridium (Boston, MA, USA) [26]. It provides an SDK, for Android and iOS devices that allows for integrating biometric authentication in any business application. Heterogeneous features coming from the camera, sensors, touchscreen, and multiple biometric factors are acquired and used to perform the authentication process. This solution ciphers the user information and stores half of the information of features in the server and the other half in the device to improve its security.

The company Aware (Bedford, MA, USA) [27] provides another product to authenticate users through behavioural and physiological biometric parameters, but not continuously as the previous ones. This solution makes use of speech, facial, dynamic keys and fingerprint recognition. Its/user product is named Knomi [28] and performs a biometric authentication through a collection of SDKs that run on the devices (iOS and Android) and on the cloud. Knomi software enables a distributed architecture on the cloud or a centralised deployment on the device.

Zighra (Ottawa, ON, Canada) [29] is another company that offers a product based on continuous authentication. Specifically, Zighra offers a platform of continuous authentication and detection of threats through the use of Artificial Intelligence for both mobile devices (with iOS and Android) and web pages [30]. One of its products, SensifyID, combines knowledge of generative behaviour models and biological systems. Specifically, this solution considers data from location, sensors, networks and user’s writing techniques. This product is based on task-based authentication techniques. It means that the user interacting with the device is asked to take specific actions to determine whether he/she is the legitimate owner.

Despite the results provided by the previous solutions, both in academia and industry there are open challenges such as the issues of adaptability, the improvement of the accuracy through the selection of significant features, and the selection of ML algorithms. Furthermore, continuous authentication systems should store and manage the users’ profiles according to the privacy regulation established by the General Data Protection Regulation (GDPR) [31].

## 3. Design of the Adaptive and Continuous Authentication System

This section describes in detail the four phases making up the design of our intelligent and adaptive continuous authentication system. In this context, the proposed system is based on the acquisition of sensors and applications usage data to model the behaviour patterns of the device owner. After examining the acquired data, we considered the use of machine learning techniques focused on the detection of anomalies. The goal of using these algorithms is to learn the usual behaviour of the device owner and detect when someone else is using the device. In the proposed solution, we have also considered the automatic and real-time adaptability to user’s new behaviours, which is a missing aspect in the majority of the related works. The previous decisions have been made by considering the restrictions of mobile devices in terms of battery, computational power and storage.

1. **Phase 0: Feature engineering.** This is a preliminary and non interactive stage where we make a first selection of dimensions and features. This initial set is subsequently refined by using feature selection techniques. It is important to notice that the whole process is done before deploying the system in the mobile device.
2. **Phase 1: Acquisition of behavioural data and dataset generation.** This phase consists of acquiring data from the mobile device and extracting the relevant features selected in phase 0. By doing it, we are able to capture the user’s behaviour and create a dataset. This dataset will be updated in real time with the new user’s behaviours.
3. **Phase 2: Computation of the authentication level.** During this stage, an ML algorithm is trained to fit a model from the user’s behaviour contained in the dataset. Periodically, the new user’s behaviour is sampled and, then, evaluated by the fitted model which returns an authentication level score.
4. **Phase 3: Automatic adaptability to new behaviours.** The last phase focuses on enabling the system adaptability through the elimination/inclusion of old/new behaviours to the dataset.

**Figure 1:**
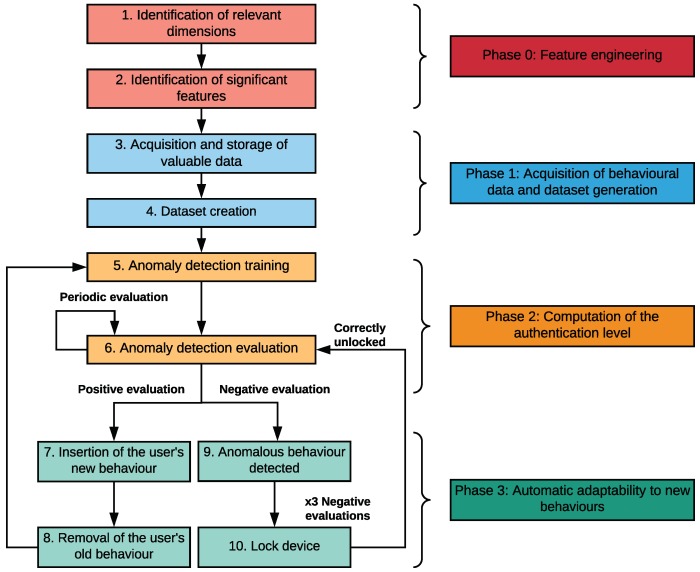
Phases and processes of the proposed adaptive and continuous authentication system.

A diagram with the different steps composing the design process is depicted in Figure 1. The rest of the section describes in detail these phases.

### 3.1. Phase 0: Feature Engineering

A critical aspect of continuous authentication systems is the selection of the dimensions that provide relevant information to model users’ behaviour (step 1 of Figure 1). Among the most well-known sources of information available in mobile devices, the most relevant are the following ones:

- *Sensors:* the accelerometer, gyroscope, or GPS are examples of sensors available in mobile devices. These sensors provide relevant information about physical movements, inclination, or geographical position, respectively.
- *Gestures and touchscreen interactions:* this dimension allows authentication systems to get relevant data such as the pressure made by users when they touch the screen, the typical gestures, the area of the screen used, or the speed sliding the fingers on the screen.
- *Application statistics:* aspects like the number of opened applications, the opening order, the time of use, or the number of applications running in the background provide relevant information about the user’s behaviour when he/she uses the mobile device.
- *Typing patterns:* the typing speed, repetition of words, number of mistypes, or expressions usage also can be used to identify users.

By considering the previous dimensions, the typing patterns of the user were discarded in this proposal due to the necessity of using the keyboard. This input method is not extensively used by the the majority of applications, and besides, when used, it supposes only a small percentage of the total device usage. In this sense, this dimension just could provide relevant information about the user’s behaviour a limited number of times [9]. On the other hand, the touchscreen interactions were not considered because they cause a high impact on the battery consumption [9]. Another drawback of this dimension is that the touchscreen interactions can be different (or ineffective) depending on the application type. For example, there are applications that just allow users to do limited actions that do not provide as relevant information as, for example, pressing a button. In contrast, other applications allow a rich variety of gestures, hence providing relevant information.

By considering the previous drawbacks, the continuous authentication system proposed in this article considers the statistics of applications as well as the sensor information as the most suitable dimensions to create a user’s behaviour profile. The statistics of applications provides useful information about the user behaviour since they can model patterns such as a common application opening order or user’s most used apps. These patterns can be used to identify the user and, therefore, to authenticate him. Moreover, it is proved that this information is commonly used in several papers and investigations, as shown in Section 2. Some papers that use this solution to classify behaviour patterns are [14,32]. On the other hand, the sensors dimension is selected because it provides information about how the user holds the device and moves it in a normal use. In addition, it is widely used in other state-of-the-art systems (Section 2). Some related papers are [8,9,15,24], where it was proved that it can model the user behaviour.

The next step was to identify significant features or characteristics belonging to the selected dimensions (step 2). For that end, we started choosing a sufficiently wide set of features, which, theoretically, might provide a differentiated modelling of user’s profiles and the subsequent authentication of the user when he/she uses the mobile device. The first experiment of Section 5 describes both the initial feature set and the process followed to select the most discriminative set, which was finally used in the Phase 1. The Table 1 lists the final features for each dimension.

Table 1 shows a new calculated coordinate called Magnitude. This value is used in [9,17] to minimise the orientation sensitivity of the inertial sensors since X, Y and Z coordinates lectures can have a negative value depending on the device orientation. Using this value allows the system to have an always positive value, not sensitive to orientation.

### 3.2. Phase 1. Acquisition of Behavioural Data and Dataset Generation

Once our system is deployed in the user’s mobile, a preliminary dataset acquisition starts. This acquisition will last for a configurable number of days (15 days in our experiments). During this time, we periodically acquire and store the raw data needed to create the user’s behaviour profile (step 3).

The majority of continuous authentication solutions that we can find in the current state of the art are based on temporal patterns. These solutions need a period of time probably longer than 15 days in order to identify patterns at different scales to model the global user’s behaviour. Nevertheless, our approach is based on the user’s statistical digital fingerprint obtained during their interaction with the mobile device. Moreover, our goal is not to model the global user’s behaviour, but their local behaviour within a given time window. Therefore, these 15 days are just an initial period of time needed to have sufficient data to allow the ML algorithm to do the first evaluations adequately. Additionally, the proposed system is adaptive; that is, it inserts each user’s new behaviour in the dataset (when the behaviour is considered as belonging to the owner). In this way, we model not only the user’s behaviour during the first 15 days, but we enrich continuously the dataset. Similarly, the user’s oldest behaviours are progressively forgotten, thus maintaining an updated window of features for a configurable period of time.

More information about the set-up of the collection process is provided in Section 4. Once the relevant data has been stored, we process them through different mathematical operations and we obtain the list of features shown in Table 1. After that, the features are grouped in vectors forming the dataset (step 4).

### 3.3. Phase 2: Computation of the Authentication Level

The main goal of this phase consists of comparing the vectors of the dataset with the current vector generated by the person that uses the mobile device in that particular moment. After this comparison, we evaluate their level of similarity and decide if the confidence in the current user is enough to consider him authenticated or not.

To perform this evaluation, it is critical to choose a suitable technique. In this sense, our solution makes use of ML techniques based on anomaly detection. The anomaly detection algorithm used during this phase is based on semi-supervised ML techniques, since all the generated vectors has the same class (user class). The first time the algorithm is trained, only owner’s samples are used and these vectors are supposed to be normal. After that, a vector generated in real time cannot be labelled since the algorithm does not know beforehand if it belongs to the device owner or to a non-authorised person. The operation of these anomaly detection algorithms is based on the existence of a dataset that reflects the behaviour of the owner. Thus, the anomaly detection method is responsible for determining in a non-intrusive way whether a current event (feature vector) fits into the patterns defined by the available dataset. The process of using these techniques has the following two stages.

- *Training.* ML algorithms implicitly maintain an internal model. In this stage the model is fitted with the updated dataset (step 5) in order to improve the accuracy of its predictions. Particularly, ML techniques applied to anomaly detection usually compute a distance measure from the normal cluster to the new sample and uses it to decide if it is anomalous or normal. Eventually, the quality, variety, and quantity of the training data, together with the complexity of the internal model determine the accuracy of the ML algorithm.
- *Evaluation.* ML algorithms evaluate the current vector of features extracted from the interaction between the user and the mobile device. This evaluation is made by means of the model fitted in the training phase (step 5), and a distance is returned indicating the degree of similarity between the current behaviour of the user and those learned by the model (step 6). If the level of similarity is greater than a given threshold, the current user is deemed to be the owner and the current vector is included in the dataset (step 7). This inclusion allows our system to ensure its automatic adaptability. Furthermore, after adding the new vector to the dataset, we perform a maintenance process to remove another one representing an old behaviour (step 8). In contrast, when the level of similarity is lower than the given threshold, the current vector is discarded due to an anomalous behaviour (step 9). If the current user generates three negative evaluations consecutively, the device will be locked (step 10), so the authentication system avoids possible attacks such as zero-effort attacks [33]. Section 5 describes in detail how the threshold was determined.

Anomaly detection techniques have been used in the cybersecurity field with excellent results, for example, to detect anomalous network traffic or to reduce the latency in 5G networks [34,35]. Nowadays, we can find different anomaly detection algorithms such as OneClass-SVM (OC-SVM) [19], Lineal Outlier Factor (LOF) [36], or Isolation Forest (IF) [37]. However, not all of these algorithms can be used in devices with computational resource constraints. Table 2 shows a comparison of the previous algorithms in terms of computational complexity. In these equations, *n* is the number of instances in the dataset, $n_{sv}$ is the number of support vector, *t*, the number of trees, *v*, the number of random samples taken from the dataset, *k* is the number of neighbours, and *d* is the dimension of the feature vector. In that sense, IF has the lowest complexity because it can be reduced to *O(n)*. It is due to the fact that *t* and *v* values do not depend on either the number of instances or the size of each instance (they have been set to 100 and 256 respectively).

In addition to the previous aspects, we have considered two main factors when choosing the proper anomaly detection algorithm for our system: training execution time and evaluation execution time. This is due to the fact that energy consumption is a critical issue in mobile devices. The ML algorithm should consume as few resources as possible because it will be periodically training and evaluating. Therefore, we have selected IF due to it has the lowest computational resource consumption among the three ML methods proposed.

### 3.4. Phase 3: Automatic Adaptability to New Behaviours

Finally, the last phase is aimed at guaranteeing the adaptability of the continuous authentication system to changes in the user’s behaviour. It is an important point of our proposal, which is missing in most of the related works. This adaptability process is performed in real time and relies on the periodical update of the dataset by inserting new behaviours and removing the old ones, whereupon the ML model is retrained:

- A new user’s behaviour is inserted in the behavioural dataset when the evaluation phase is positive, that is, the vector received an authentication level score indicating that it belongs to the owner, or it is an acceptable deviation (step 7). This aspect allows our continuous authentication system to learn a new user’s behaviour.
- The vectors associated with old behaviours will be progressively discarded as new vectors are inserted to assign more importance to new user’s behaviours (step 8). This fact allows our solution to ensure the *right to be forgotten* as well as to avoid an increasing execution time in training and evaluation due to the size of the dataset.

## 4. Deployment

This section is devoted to the implementation details of the proposed system. In this context, we have designed and implemented a mobile application for the Android operating system that implements our adaptive and continuous authentication system. The acquisition of some important features, defined in Phase 0, is possible only with Android 5.0 (Application Programming Interface, API, level 21) or newer. However, this requirement is fulfilled by more than 80% of mobile devices [39].

**Figure 2:**
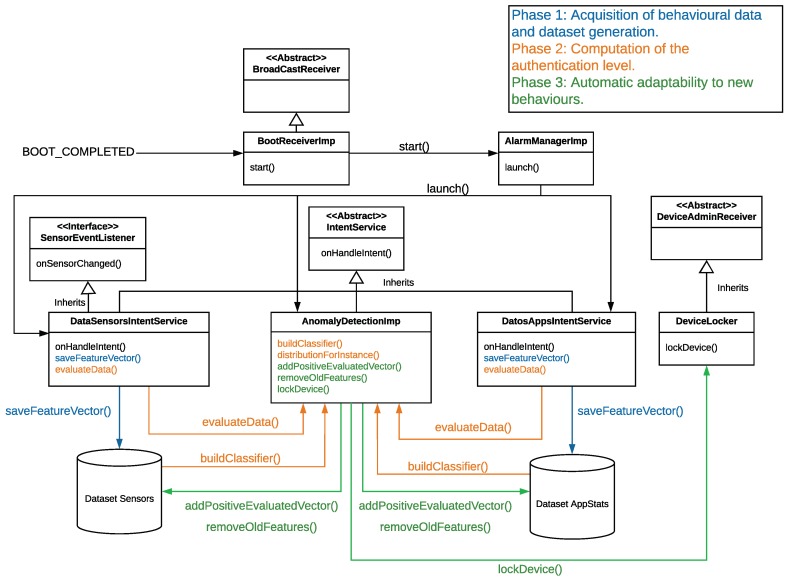
Diagram of the most important classes and processes of the system.

Figure 2 depicts a diagram of the most relevant classes making up our mobile application. These classes implement the functionality described in Section 3. The collection services used to acquire the user’s behaviour and create the dataset (Phase 1), the training and evaluation of the ML algorithm (Phase 2), as well as the maintenance of the dataset adding and removing new behaviours (Phase3), are some parts of the diagram that deserve an special mention. For clarity’s sake, we have drawn the elements of Figure 2 using the same colors as the phases defined by Figure 1).

With the goal of carrying out the acquisition of relevant data from sensors and applications (the step 3 of Phase 1), we have implemented the following classes and methods. Each class is tagged with the value *(A)* when it belongs to the Android libraries, and the value *(O)* when it is one of our implemented classes:

- *BootReceiverImp (O)*. This class is the starting point. It inherits from BroadcastReceiver [39], which belongs to the Android library. BootReceiverImp receives an event when the mobile device is turned on and configures the alarms needed to execute the functionality of our mobile application, even when the application is not running.
- *AlarmManagerImp (O)*. Its functionality focuses on reducing the consumption of battery by executing the services periodically and not continuously. The *launch()* method is in charge of scheduling periodically the data collection services using the *setExact()* method from AlarmManager class [39].
- *IntentService (A)*. This class allows the execution in background of the needed operations to obtain the data from which the features will be computed.
- *DataAppIntentService (O).* This class utilises the *Android.app.usage* class [39] to gather data about the applications usage. The *onHandleIntent()* method is responsible for the periodical collection of the application data and the calculation of the related features. Every 60 s, if the device is unlocked, this method gathers the list of applications used in that minute, ordered by the moment in which each app was put in foreground; the opening sequence; and the time of use of each application since there are statistics. After that, it carries out different operations to obtain and calculate the application features enumerated in Table 1.
- *DataSensorsIntentService (O).* This class obtains data coming from the device sensors. It implements the *Android.hardware.SensorEventListener* interface, which is used to receive notifications from a variety of sensors. The *onHandleIntent()* method periodically acquires data coming from different sensors. Specifically, every 20 s, if the device is unlocked, this method reads the X, Y and Z sensors (accelerometer and gyroscope) values for 5 s. Then, a moving average with a window of three samples is applied to these values, having an effect similar to a low-pass filter and, hence, reducing the measurement error. These filtered values are then aggregated into the sensor features show by Table 1.

After obtaining periodically the features, the next step is to generate the dataset that models the user’s behaviour. In this context, the DataSensorsIntentService and DataAppIntentService classes have a common method, *saveFeatureVector()*, in charge of saving periodically the features in two different datasets. One for the sensors’ features, and another one for the applications. In this point, it is important to clarify that the datasets are generated after monitoring the user’s behaviour for 15 days. This period of time has been defined after performing several experiments with different time duration, concluding that it is an acceptable time to obtain accurate authentication results.

Once the dataset has been created, Phase 2 starts (step 5 of Figure 1). In this phase, we used the implementation of IF provided by the Weka library [40]. Our mobile application implements the *AnomalyDetectionImp* class, which uses some of the methods provided by the Weka library to train and evaluate IF; specifically, the *buildClassifier()* and *distributionForInstance()* methods, respectively.

Regarding the training phase, we use the *buildClassifier()* method to build two IF: one for detecting anomalies in the sensor dataset, and another for detecting anomalies in the application dataset. The configuration parameters set for the IF training are shown in Table 3.

The first training of our models is performed after finishing the Phase 1 (15 days) with the aforementioned dataset containing feature collection. Each of them uses one different dataset: sensor dataset and application dataset. From that particular moment, in this proof of concept, the IF algorithms are trained separately every positive evaluation, but it can be configured according to the requirements of the scenario.

On the other hand, the evaluation of the new two feature vectors (sensors and apps) are also carried out separately and every 60 s, which coincides with the acquisition frequency of feature vectors. However, it could be different, and we could reduce the frequency for energy consumption restrictions. To reach that goal, the *AnomalyDetectionImp* class executes the *distributionForInstance()* method for both IF algorithms, receiving as parameters the corresponding vector to be evaluated. As a result, we obtain the scores of the two current vectors of features (sensors and applications), that is, a number between 0 and 1 (where 0 means highly anomalous and 1 completely normal). The configuration parameters, considered to evaluate the IF algorithm, are shown in Table 4.

Once evaluations for sensors and applications are performed, the scores should be normalised before combining them to get a unique and final score. This score will represent the authentication level of the user interacting with the device in that particular moment. In this sense, a min-max normalisation is required because each evaluation is computed by a different IF algorithms, resulting in output values belonging to different ranges of the interval [0,1]. The normalisation expression can be found in Equation (1), where *NS* is the normalised score, *x* is the current evaluated vector value, and *max, min* are the maximum an minimum values obtained during the evaluation of the dataset:

(1) $$NS=\frac{\left(x-min\right)}{\left(max-min\right)}.$$

After normalising the two evaluations, Equation (2) shows the next step, which consists in combining and calculating the final authentication level score:

(2) $$AL=Las\cdot Wa+\frac{Lss+Pss}{2}\cdot \left(1-Wa\right).$$

In the previous equation, *AL* is the Authentication Level score [0,1] calculated by our solution to indicate how similar the behaviour of the current user is compared to the owner. *Las* is the classification score of the last application vector (normalised), *Lss* is the classification score of the last sensor vector (normalised), and *Pss* is the classification score of the penultimate sensor vector (normalised). *Wa* is a constant parameter that indicates the weight or importance of the last application vector in the final result. In this case, *Las* has the value of 0.5 because we assumed that sensor and application dimensions have the same relevance to determine whether the behaviour of the current user belongs to the owner of the device. As previously, this parameter can be modified according to the scenario characteristics.

If the AL score is higher than a given threshold, which has been previously defined by us (explained in the Section 5.1.5), the user is positively authenticated by the proposed system. In this case, the *AnomalyDetectionImpIF* class has two different methods in charge of providing adaptability to new user’s behaviours in real time. On the one hand, the *addPossitiveEvaluatedVector()* method inserts the new vectors of features in both datasets (sensors and applications). On the other hand, the *removedOldFeatures()* method removes old vectors of features stored in both datasets. However, if the AL score is lower than the threshold, the user is not authenticated and the datasets are not updated. Furthermore, if the AL is not over the threshold after three consecutive evaluations, the *lockDevice()* method will be called, which uses *DeviceLocker* class to lock the device screen. This class inherits from *Android.app.admin.DeviceAdminReceiver* [39] and uses the *Android.app.admin.DevicePolicyManager* [39] class functionality to lock the device. As said in Section 3, this action is performed to prevent a zero-effort attack.

## 5. Experiments

This section shows different experiments carried out to validate the viability of our intelligent and adaptive continuous authentication system. Specifically, to design and implement our system, we have performed a pool of experiments related to the accuracy of the system, its adaptability to changes in the user’s behaviour, its resiliency against adversarial attacks, and its resource consumption in terms of energy, storage, and time.

### 5.1. First Experiment: Feature Engineering and Anomaly Detection Performance

This first experiment is part of the aforementioned Phase 0 and it focuses on the selection of relevant features, as well as the determination of the threshold used to distinguish between anomalous and normal behaviours. To reach those goals, the experiment has been carried out by using the preliminary dataset obtained in stage 0. Its aim is to extract the most discriminative features from the initial feature set (listed in Table 5), train two IF algorithms with each set of features and test whether the selected set has a better classification performance by means of precision vs. recall AUC (Area Under Curve) measure. After that, a suitable threshold to separate normal from anomalous samples was determined for the winner model in order to obtain an estimation of the classification performance reached. Finally, the suitability of the previous threshold has been validated with 50 different users.

#### 5.1.1. Data Collection

Table 5 lists the set of features used to obtain our initial dataset from two different users: *UserA* (the owner) and *UserB* (an unknown person). To perform this experiment, the owner of the mobile device, UserA, trained our authentication system for 15 days. Once this process was finished, we created one dataset with all the application usage features, and another with all the sensor features. In the case of the application usage dimension, vectors were generated every 60 s when the device was not locked. As a result, once the training was completed, the owner had generated 8700 vectors (780 KB). In the case of sensor dimension, vectors were acquired every 20 s when the device was not locked. The total number of vectors generated by this dimension is 13,800 (4 MB). Both datasets were labelled as normal. After that, a new user, UserB, started using the UserA’s mobile device. The user data was collected for five days, generating two new datasets labelled as anomaly.

#### 5.1.2. Feature Selection

Since dimensionality reduction usually provides an improvement in the detection of anomalies [41,42], a selection of the most relevant features was performed. First, after carrying out a variety of tests and analysing their results, we concluded that the data acquired from the magnetometer sensor were erratic, since the values depend on the geographical orientation of the device. After that, we decided to do feature selection by using a random forest in order to obtain a subset of the features with the most discriminating properties.

For this procedure, an auxiliary labelled and balanced dataset was created by combining both application and sensor information from UserA and UserB. The new vectors were created concatenating every vector from the sensor dataset with the corresponding application vector closest in time. The resulting dataset was then labelled accordingly (UserA’s samples as normal and UserB’s as anomaly), and the random forest was trained.

One interesting property of random forest is that, after training, it provides an estimation of the discriminating power of each feature during the classification process. These values represent the relative weight or importance of each feature in the decision [43]. These coefficients were used to select a subset of features that covered 95% of the total relative weight. The selected subset was already presented in Table 1, whereas the initial feature set can be found in Table 5.

#### 5.1.3. Application and Sensor Datasets’ Aggregation

Once we have the two sets of characteristics, the next step is to combine the relevant features of each dimension to evaluate the user’s behaviour and detect anomalous patterns using the IF algorithm.

Only one IF would be necessary for anomaly detection. However, we realised that the performance was improved if a specialised IF was trained with each dataset (applications and sensors). After training, a sample is then classified by obtaining the anomaly score from each IF, averaging the two scores, and using this result together with a decision threshold to predict whether the sample is normal or anomalous.

The output of our model had to combine the outputs of the two aforementioned IFs, but each output had a different range. To make them compatible, the scores from each IF in the training stage were normalised to the range [0,1]. Then, they were averaged to obtain a single value of anomaly estimation. This procedure was done twice: once with the initial set of features and the other one with the set selected by means of the random forest. As a result, we obtained two models to be compared in order to decide which had a better performance.

#### 5.1.4. Performance Comparison for Feature Set Selection

In order to determine whether the new feature set had a better performance than the initial set, we needed to obtain a measure of the performance for every threshold. In our case (an anomaly detection context), the dataset is highly unbalanced, making the accuracy meaningless. In this case, a more suitable estimator is the precision vs. recall AUC. The next equations define a classification algorithm, the concepts of precision (3), recall or TPR (True Positive Rate) (4), and F1-score (5), which combines precision and recall in a single value for comparison purposes:

(3) $$\mathrm{Precision}=\frac{\mathrm{True}\;\mathrm{Positives}}{\left(\mathrm{True}\;\mathrm{Positives}+\mathrm{False}\;\mathrm{Positives}\right)},$$

(4) $$\mathrm{Recall}\;\mathrm{or}\;\mathrm{TPR}=\frac{\mathrm{True}\;\mathrm{Positives}}{\left(\mathrm{True}\;\mathrm{Positives}+\mathrm{False}\;\mathrm{Negatives}\right)},$$

(5) $$F1-\mathrm{score}=\frac{2\times \mathrm{Precision}\times \mathrm{Recall}}{\mathrm{Precision}+\mathrm{Recall}}.$$

In other words, the precision tells us how good our classifier is detecting real anomalies, that is, the higher the precision, the lower the false alarm percentage; recall indicates how good it is recognising all the real anomalies, that is, the higher the recall, the fewer anomalies remain undetected; finally, F1-Score is the harmonic average of precision and recall. F1-score allows for summarising in one value both precision and recall, making the comparison of results easier.

**Figure 3:**
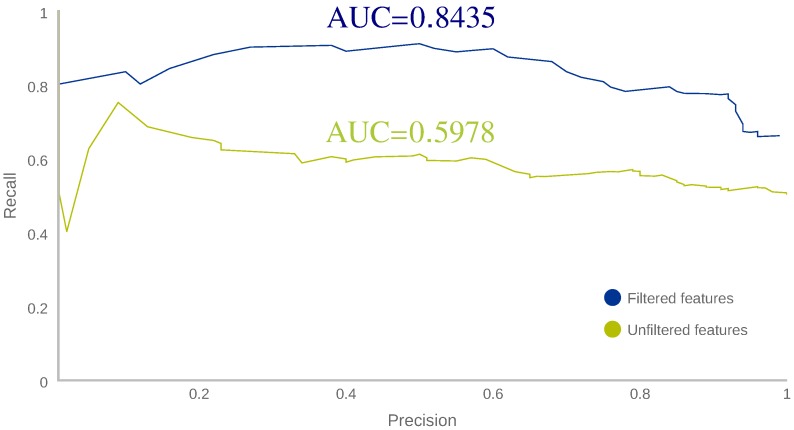
Area under the curve (AUC) of our model trained with both the initial and selected set of features.

Figure 3 plots both precision vs. recall curves and shows that the selected feature set has a greater AUC. It can also seen that it has better classification performance for every threshold since filtered features line (blue) is always over the unfiltered features line (green); therefore, the set of selected features was chosen for the next step.

#### 5.1.5. Determination of the Optimal Threshold

**Figure 4:**
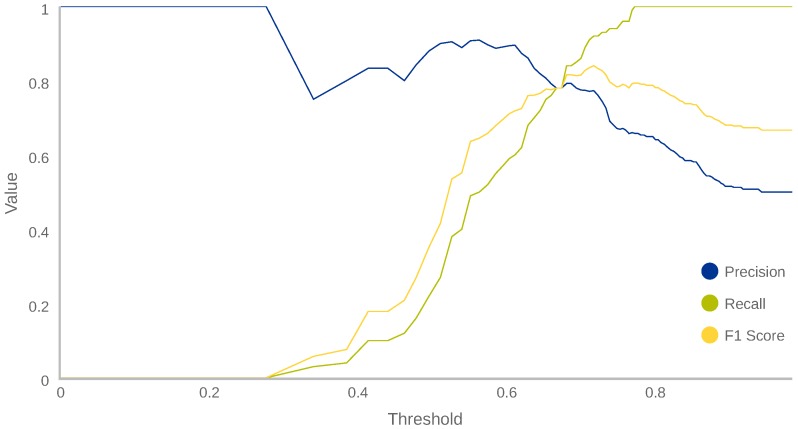
Recall, precision and F1-score vs. threshold when our model is trained with only the selected features from UserA’s dataset, and evaluated on users A and B.

In our anomaly detection context, a sample is considered normal if its classification score is over a given threshold; otherwise it is considered anomalous. In order to reach an optimal classification performance, we need to determine the optimal threshold. Figure 4 depicts three curves: precision, recall and F1-score vs. threshold. The figure shows how, when the threshold is very low (less than 0.30), the precision is 1 (every normal known behaviour is evaluated as normal) and recall is 0 (every anomalous known behaviour is evaluated as normal). In the same way, when threshold is too high (more than 0.93), the precision value is 0.50 and recall is 1 because every behaviour evaluated will be taken as anomalous, even the known normal ones. Thereby, threshold should be established at an intermediate value that provides the best performance. Here, the chosen threshold will depend strongly on our requirements. A common selection criterion is the maximum F1-score (0.84 in this case, corresponding to a precision of 0.77 and a recall of 0.92), resulting in a threshold of 0.728. In our case, we preferred this last threshold because the highest F1-score guarantees a good balance between precision and recall, without prioritising any of them.

#### 5.1.6. Anomaly Detection Results

Once the threshold was selected, our system was tested with UserA and UserB. Table 6 shows the confusion matrix with the classification results of a test set with 200 new samples (100 normal samples from UserA and 100 anomalous from UserB) together with the precision and recall.

**Figure 5:**
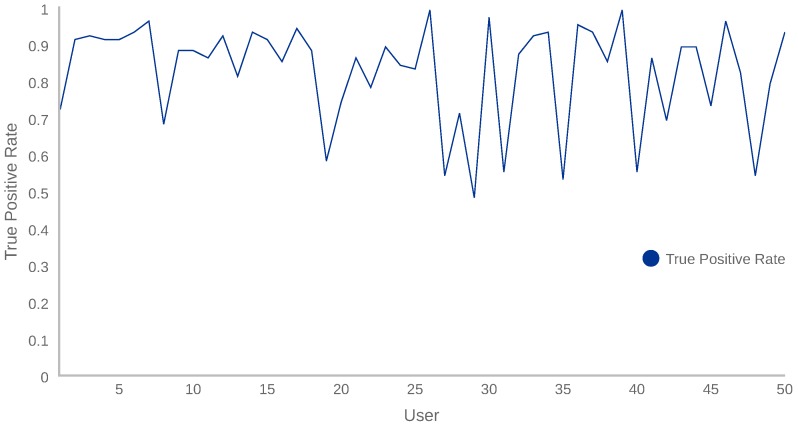
True positive rate for the 50 evaluated anomalous users.

In addition, we performed some additional tests to validate the model and demonstrate that our continuous authentication system could accurately classify as anomalous the behaviour of users others than UserB. In this sense, fifty new users, who had not used the device during the training phase, started using our solution. The TPR measurements obtained after evaluating 100 samples from each new user (anomalous behaviour) are shown in Figure 5.

Figure 5 shows how TPR measurements of the fifty users range from 48% to 98%. This variability indicates that, depending on the users’ behaviour, the proposed system recognises the anomaly more or less accurately. By analysing the results, we realised that the users interacting with the mobile device lying on a table obtained a lower TPR. This is motivated by the fact that the owner sometimes operated the device on a table, resulting in almost constant sensor measurements and, hence, evaluations with a higher AL score. Additionally, it is important to notice that the mean TPR of all evaluations is 82%, which is aligned to the results shown in Table 6, where the TPR is 92% and the True Negative Rate (TNR = TN/(TN + FP)), associated with the performance of the device owner, is 73%.

In conclusion, the proposed solution is able to distinguish the normal user’s behaviour (UserA) among another 50 anomalous users with satisfactory performance. Regarding the recall value, it is 92% and the precision is 77%.

### 5.2. Second Experiment: Adaptability to New Behaviours

This experiment demonstrates how the proposed system is able to adapt itself to changes in the user’s behaviour in real-time. For that end, our model is based on the combination of two IF algorithms (sensors and applications) trained with selected features stored in the sensor and application datasets, respectively. The output of our model is called AL (Authentication Level) and its expression can be found in Equation (2). In order to make our system adaptive, we propose the partition of the AL score in four segments, meaning respectively: certainly anomalous, possibly anomalous, possibly normal and certainly normal. To determine the range of values of each segment, we based our decision on the precision of the model obtained in the first experiment. Once the four ranges are established, our system uses them to decide when and how to adapt to a change in the user’s behaviour. Every 60 s, our system uses the ML model to generate a new AL score from the application and sensor information, summarising the behaviour of the user during that period of time. The actions that our system takes to adapt itself depend on the score segment to which the value belongs. When new vectors are added, the oldest vectors will be discarded; therefore, the dataset will always contain a fresh set of user’s behaviours. The rest of the section describes in detail this experiment.

#### 5.2.1. Certainty Classes

**Figure 6:**
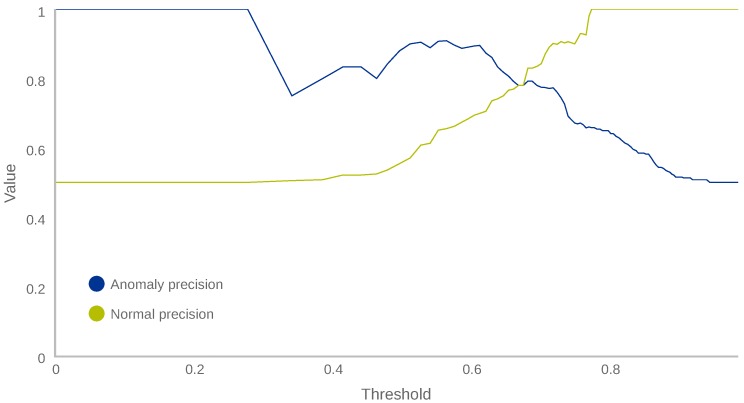
Precision curves on both normal and anomalous behaviours.

Our first step was to define the four score segments. To this end and using the behaviour dataset collected from UserA and UserB, the classification precision that our model gives for each label (“normal” and “anomaly”) was computed for each possible threshold. Figure 6 plots the two precision curves obtained.

The curves of Figure 6 show the collected behaviour vectors evaluated by the model, obtaining an AL score, which was used to classify them with respect to a given threshold. If the AL value is lower than the threshold, the vector is classified as anomalous; otherwise, the vector is considered normal. The precision curve of the “normal” label in Figure 6 shows that every vector with an AL score above 0.78 is correctly classified as normal. Similarly, the precision curve of the anomaly label shows that every vector with an AL score below 0.35 is correctly classified as anomalous. Additionally, there is a value where both curves cross, that is, 0.67. Below this value, the number of samples classified as anomalous exceeds the number of samples classified as normal, and above this value the opposite is true. Table 7 shows the different certainty classes established.

#### 5.2.2. Adaptability

Once the previous thresholds were defined, we evaluated how the AL of a vector changes when a set of vectors associated with similar behaviours are inserted in the dataset. Instead of forcing the owner of the mobile to repeat approximately the same actions with the apps for hours, we created two sets made up of variations of a user’s vector (one set for the sensor dataset and another for the application dataset) by adding some noise. In the case of the sensor vector, where the values are float, a random noise obtained from a normal distribution with mean 0 and variance 0.05 was added. In the case of the application vector, the noise was obtained by sampling a uniform distribution between 0 and 1. For each integer attribute composing the vector, we subtracted 1 if the random sample was below 0.2. In contrast, when the random sample was above 0.8, we added 1 to the integer value.

With these tests, we tried to figure out how many times it would be required to introduce a vector with similar features to produce an adaptation of the authentication system to the new behaviour. At the same time, the old vectors representing outdated user’s behaviours would be removed. This adaptation would happen, for example, if a new application was installed on the mobile, or the user operates the device with the unusual hand due to an injury.

The aim of our first test was to demonstrate the adaptability of the system to changes in the application dimension. The action studied was the installation of a new application in the user’s mobile. After the installation, the mobile device was used in a usual way; therefore, only the application dimension would be affected by this change.

**Figure 7:**
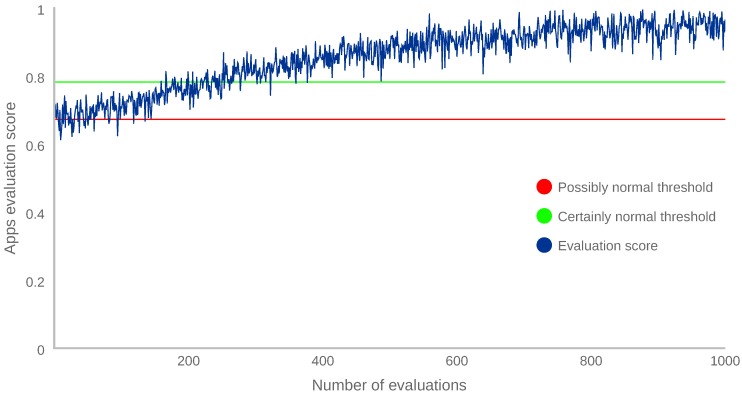
App evaluation scores of the new application vectors as the number of similar new vectors increases.

A set of 1000 vectors was generated by adding noise to a vector collected from the owner while using the mobile device with the new application. Each generated vector was evaluated by the IF algorithm, and added to the dataset (removing the oldest vector) if the score belonged to the ranges possibly normal or certainly normal. After each insertion, the model was trained again. This was repeated for each vector in the set. Figure 7 shows how the normalised evaluation score for applications, Las in Equation (2), increases when vectors are added to the dataset.

**Figure 8:**
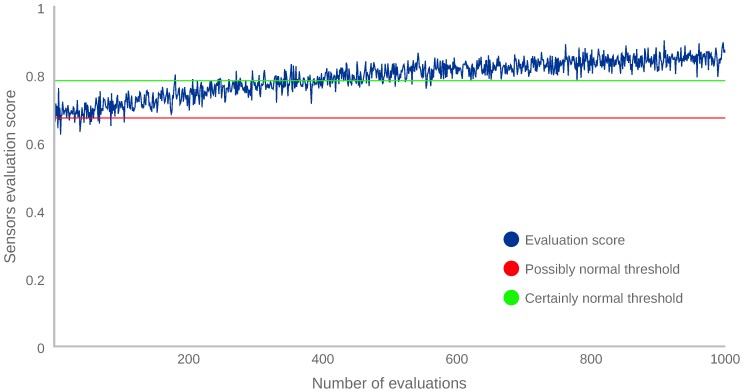
Sensor evaluation scores of the new sensor vectors as the number of similar new vectors increases.

Similarly, the same procedure was performed using a new sensor vector representing a behaviour change. In this case, Figure 8 shows how the sensor vector evaluation score, Lss in Equation (2), increases when new vectors are added to the dataset.

Figure 7 and Figure 8 depict that, when a family of new vectors is introduced in the dataset, the evaluation scores progressively increase, eventually changing the evaluation score of those vectors to certainly normal.

Finally, after performing the experiment and observing the results, we can conclude that the proposed continuous authentication system is able to adapt itself to a new behaviour in about 300 iterations, which is equivalent to 5 h of device usage for this particular case. The main reason for that duration is due to the fact that IF needs a significant number of similar vectors to start scoring them as normal. This could be improved by increasing the number of random samples. However, this number usually is hard-coded in the machine learning libraries. Specifically, each IF is configured to construct 100 decision trees by using only 256 randomly sampled vectors from the dataset. The way IF scores a vector is by averaging the scores from each decision tree. Then, a new vector is included in the training process only if it is selected in this random sampling. In addition, this vector has to be frequent enough to be randomly included in several trees and to have an impact on the final score. Therefore, the adaptability time is directly related to the number of similar instances of one vector contained in the dataset.

### 5.3. Third Experiment: Adversarial Attacks

This experiment shows how the proposed intelligent and adaptive continuous authentication system reacts to two adversarial attacks. In this sense, we defined a scenario where the owner of the device spent two weeks interacting with the device. During these two weeks, phase 1 of the proposed solution was executed, acquiring data from sensors and applications and generating a dataset. Once phase 1 was finished, our system trained the IF algorithm with the previous dataset. After that, we prepared the following two adversarial attacks:

1. Trial and error attack. The mobile device was operated for 10 min by five attackers who were not aware of the owner’s behaviour.
2. Shoulder surfing attack. The device was operated for 10 min by five attackers who had seen the owner’s behaviour in terms of position and applications usage for five minutes.

For each one of the previous attacks, we evaluated the vectors (attempts) generated by the attackers during the 10-minute interaction with the mobile device, and analysed the AL provided by our solution.

**Figure 9:**
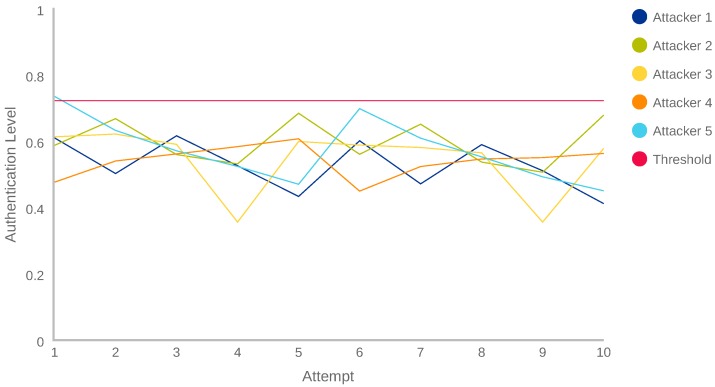
Trial and error attack scores.

Figure 9 shows the AL results obtained by the five attackers during the trial and error attack. As it can be seen, 49 of the 50 attempts (10 per each one of the 5 attackers) obtained an AL value lower than the 0.728 threshold calculated in the Experiment 1 (Section 5.1). It means that they were detected as anomalous users or attackers. Here also, it is important to note that the majority of the evaluations are between the values 0.4 and 0.65 (established as possibly anomalous by Table 7).

The results of Figure 9 demonstrate that, when the user’s behaviour is not known, the system obtains a high accuracy detecting anomalies. Another important point to note is the fact that two of the attackers (Attacker 5, attempt 6 and Attacker 6, attempt 5) obtained an AL value higher than 0.68, our adaptability threshold. This fact can occur due to a random similarity between the attacker and the owner. Anyway, as it is indicated in Table 7, the values comprised between 0.67 and 0.78 represent that possibly it is the owner, but the system does not have the total certainty.

**Figure 10:**
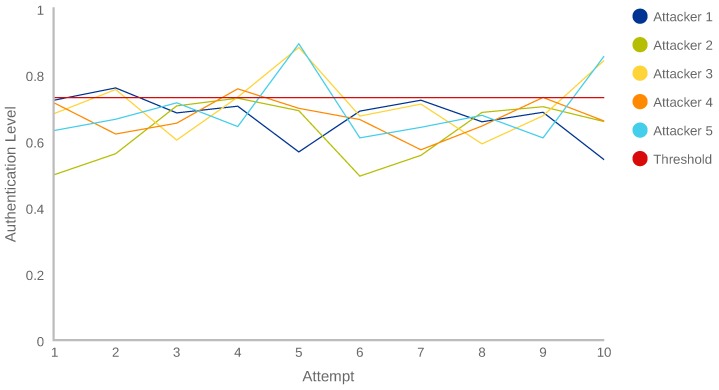
Shoulder surfing attack scores.

On the other hand, Figure 10 shows the AL results obtained by the five attackers of the shoulder surfing attack. As can be seen, the AL values are higher than in the trial and error attack. In this case, there are 9 of 50 attempts with an AL score higher than the authentication threshold (0.728), which means that they were authenticated as the owner. Another important aspect to highlight is that the majority of the AL scores are comprised between 0.6 and 0.7. That indicates that they could be normal (see Table 7).

After analysing the results of both attacks, when an attacker is aware of the owner’s behaviour he/she obtains higher AL scores than if not, as expected. However, the proposed system is able to detect correctly 98% of trial and error, and the 82.5% of shoulder surfing attacks. These percentages are completely aligned with the results obtained in the Experiment 1 (Table 6), where the precision for anomalies was 77%, and the recall was 92%.

In conclusion, this experiment has demonstrated the resiliency of the proposed solution to adversarial attacks such as trial and error and shoulder surfing. The main reason why our solution is robust to these adversarial attacks is the correct selection of features. On the one hand, regarding the application dimension, there are some features that cannot be learned by attackers when they look at the owner’s behaviour. Among these features, we highlight the number of apps opened during the last minute, or the application opening order. On the other hand, regarding the sensor dimensions, although attackers can learn the position in which the owner uses the device, they cannot duplicate the vibrations, inclination and orientation of owner’s device in specific moments. In this sense, the inclination and orientation alter the mean values, and the vibrations affect the maximum, minimum and variance of the accelerometer and gyroscope. Additionally, the fact that attackers do not know either the exact moment when the evaluation is performed or the evaluation frequency increases the level of difficulty to copy the owner’s behaviour. Finally, it is also important to note that the proposed system locks the mobile device when the AL score is lower than the predefined threshold (0.782) three consecutive times. Nevertheless, to obtain accurate results in this experiment, we have disabled this security mechanism.

### 5.4. Fourth Experiment: Resources Consumption

The experiments shown in this section focus on measuring the energy, storage, and time consumption of the proposed system. For that, we have been performed different tests in the two following mobile devices: Huawei P10 Lite, 3300 mAh battery, Android 7.0; and Xiaomi Redmi Note 4 Pro, 4180 mAh battery, Android 6.0.

#### 5.4.1. Energy Consumption Experiment

Energy consumption is one of the most important concerns of continuous authentication systems for mobile devices. In this sense, we performed the following energy consumption experiment to calculate the impact of our system on the battery life of two different mobile devices. The main goal of this experiment was to measure both the battery percentage and the total amount of milliampere hour (mAh) consumed by our solution in a complete life cycle.

In this experiment, we monitored the battery consumption when the mobile devices were used in a normal way during the evaluation phase. In this phase, the data collection and evaluation services were running every minute (having the device unlocked) and every 20 s, respectively (as explained in Section 4).

To obtain the results shown in Table 8, the experiment was repeated 10 times in each device averaging the measurements. As an illustration, the Xiaomi device had our continuous authentication system running an average of 10 h per day, until the battery became drained. The total energy consumed by our system was 293 mAh, that is, 7% of the total battery capacity (4180 mAh). As can be seen in Table 8, the Huawei device behaved similarly.

By taking into account the previous results, we conclude that the energy consumption of our solution is acceptable because it does not penalise, in a significant way, the normal functioning of the mobile devices and, therefore, the users’ quality of experience.

#### 5.4.2. Storage Consumption Experiment

Storage consumption is another key aspect of mobile devices due to their limitation in terms of resources. In this sense, we carried out an experiment focused on calculating the storage required by our intelligent and adaptive continuous authentication system. Specifically, Table 9 shows, for two different devices, the size and number of vectors of the datasets generated after a collecting period of 15 days. At this point, it is important to note that, during the collecting period, both devices were used in the usual way.

As can be observed in Table 9, for both devices, the storage consumption of the application and sensor datasets is less than 1% of the device available storage, which is negligible. This fact is rather positive because it enables our continuous authentication system to work without storage concerns in practically any device.

#### 5.4.3. Time Consumption Experiment

Finally, time consumption is another critical aspect for a continuous authentication system operating in real time, such as the proposed in this article. In this sense, the last experiment focused on measuring the time required by our solution to decide whether the current user is the owner of the device, or not. With that goal in mind, we measured the time required by our proposal to perform the training and evaluating processes. This was repeated 10 times. The averaged results are depicted in Table 10.

The times shown in Table 10 are rather acceptable since the training and evaluation processes of both dimensions (applications and sensors) are executed in less than five seconds in both devices. In this sense, to improve these times we propose to train the IF algorithm less frequently, instead of every dataset update (frequency used in our proof of concept). The training process could be done at the beginning of every day or every a given number of hours (e.g., the five hours needed to incorporate a new behaviour in our second experiment). The evaluation process can be considered negligible (1 ms).

## 6. Discussion

This section discusses and compares the main characteristics of the most relevant solutions presented in Section 2 with the adaptive and continuous authentication system proposed in this article.

The solution presented by Bo et al. in [8] uses typing patterns and sensor data to classify the user’s behaviour, by means of both a semi-supervised (OC-SVM) and supervised (SVM) methods. In contrast to our solution, this proposal does not propose any technique to enable the system adaptability to new behaviours. Moreover, the paper does not include a discussion of the training and evaluation complexity.

Ehatisham-il-Haq et al. [9] presented a system to identify the interaction of users with their devices based on the use of the accelerometer, gyroscope and magnetometer. The authors combine a k-means clustering and a classification method to obtain an anomaly score. Several ML classifiers are assessed, and eventually the SVM algorithm is selected, providing high accuracy (99.18%) at the expense of a excessive computational cost. The power consumption is not analysed; however, the authors recommend algorithms such as Bayes Net or Decision Tree which need less resources with just a small decrease on accuracy to be used with smartphones. In addition, the system does not take into account the possibility of changes in the user’s behaviour over time. This work provides information about the execution time of the evaluation: 2.32 s for k-NN, 5.61 s for Bayes Net, 10.11 s for Decision Tree, 25.21 s for SVM. In overall, Bayes Net shows the best performance in accuracy/time execution.

The proposal presented by Fridman et al. in [14] acquired features such as location, applications usage, text typed by the user and visited websites to later combine the results obtained from the classification process. In this paper, the location is the dimension with more relevance to classify the user’s behaviour. However, this dimension has a great impact in the battery consumption, which is critical in the mobile device context. Additionally, if a malicious user operates the mobile in the same places as the owner, the authentication process will fail. Finally, like the previous proposals, this solution make use of supervised classification algorithms to determine the user’s behaviour and its solution does not consider the possibility of adaptation to new behaviours.

Another interesting authentication system is proposed by Centeno et al. in [15]. This proposal obtains information about the sensors of the device to detect anomalies in the user behaviour. This solution has a high computational cost since Autoencoders [44] are used to perform the authentication process. This fact provokes that training is carried out in the cloud, with the privacy concerns associated with the handling of sensitive information. Additionally, another limitation of this proposal is that, to ensure its proper functioning, the mobile device must be connected to the internet.

The proposal of Li et al. [17] obtains excellent results in terms of accuracy, False Acceptance Rate, False Rejection Rate and Equal Error Rate. However, their proposal is not adaptive. In addition, the energy consumption of their system is higher than the consumption of ours. On the other hand, the work proposed by de Fuentes et al. [21] performs a user classification based on non-assisted sensors achieving 97% of accuracy using only battery reading information. When the system tries to identify both user and environment by combining data from different sensors, like battery readings, ambient light and ambient noise sensors, it obtains 81.35% of accuracy. The algorithm that provides the best results in this case is k-NN. However, this solution does not take into account the adaptability of the system.

The Veridium product [26] authenticates the user by means of biometric information. This solution splits the vectors of features acquired into two sets. One of them is stored in cloud servers, and the other is stored in the device. Therefore, the mobile devices must always have an active internet connection. If the internet connection is down, the mobile will not be able to access to both parts and, therefore, it will not be able to evaluate the user. As it is a market product, it is not specified what type of ML technique has been used. This product is designed for authenticating the user based on biometric information, but it does not work as a continuous system that evaluates the user periodically.

The company Aware [27] provides a market product that combines behavioural and physiological parameters to authenticate users. The main limitation of this product is that it only authenticates the user at specific times; that is, the product does not perform a continuous authentication of the user. Although this proposal makes use of some well-known dimensions, this product can not be compared with our system because it is not a continuous authentication solution.

Finally, the company Zighra [29] provides its product known as SensifyID. SensifyID uses its own Artificial Intelligent algorithm, which runs in the cloud and it is not public. Due to the private nature of the solution, it is not possible to compare it with ours in terms of accuracy and resource consumption. Zighra claims that its AI is able to quickly learn the user’s behaviour (about 15 interactions). This proposal requires an active internet connection to perform the authentication process, which is an important drawback depending on the use scenario.

Except the work of Ehatisham-il-Haq et al. [9], which provides information about the execution time, and the work of Li et al. [17], which provides information about time efficiency and energy consumption, the rest of papers and commercial products do not provide information about time, storage or energy consumption. We consider this information relevant to determine whether the proposed system meets two basic requirements for being executed on mobile devices. First, the proposed continuous authentication system should have a low power consumption, since battery power is limited. This restriction is related to the execution time and is the reason why it is preferable to choose a faster method even if its accuracy is slightly smaller. Secondly, the size of the dataset in memory should be as small as possible because memory can be a scarce resource.

As can be seen in Table 11, our proposal improves some of the limitation of existing solutions. To reach it, our solution combines two dimensions (the application statistics and the sensors of the device) with a semi-supervised anomaly detection algorithm to perform the authentication process. In addition, the proposed system is adaptive and detects changes in the user’s behaviour without forgetting the resource consumption limitations of mobile devices. Moreover, the proposed continuous authentication system is also self-contained in the device, so an active internet connection is not necessary.

## 7. Use Case: Online Banking Mobile Application

**Figure 11:**
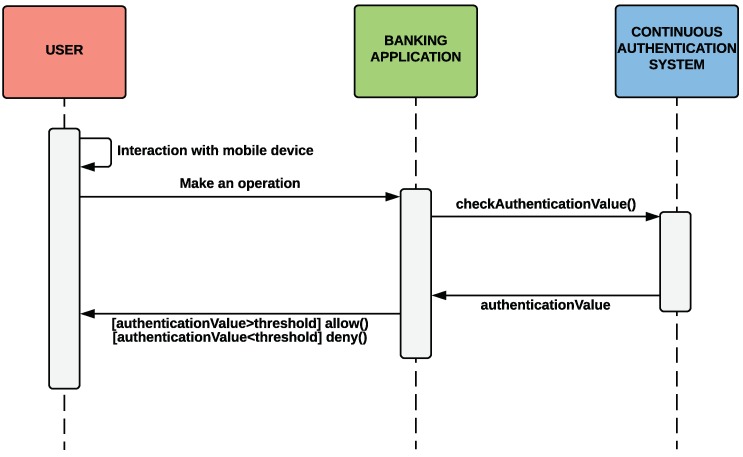
Flow diagram of the use case.

This section shows a use case that highlights the utility of our continuous authentication system in a relevant scenario such as an online banking mobile application. In this context and as proof of concept, we have designed and implemented a banking mobile application for our *Nevele Bank* MOCK [45]. This mobile application implements the functionality of the Nevele bank [46] by allowing users to see the status of their bank accounts, make transfers with different amounts of money, and so on. Additionally, our banking application is able to communicate through an API with our continuous authentication mobile application explained in Section 4. The fact of integrating the banking mobile application with our continuous authentication system allows the user to minimise the use of authentication credentials. Therefore, the banking application will allow the user to perform certain operations or not depending on the AL score returned by our adaptive and continuous authentication system. The interaction sequence between the user, the banking application, and our continuous authentication system is shown in Figure 11.

To perform the tasks allowed by the online banking application, we have established four thresholds related to the AL score returned by our system in real time (see Table 12). To determine the thresholds, we have used the information that provide the experiment performed in Section 5.2.

At this point, we have performed different experiments to show the utility of our solution in a banking scenario. For that end, our authentication system has been trained for 15 days by the UserA, and we show the cases identified by Table 12.

### 7.1. The Banking Application Cannot Be Opened

**Figure 12:**
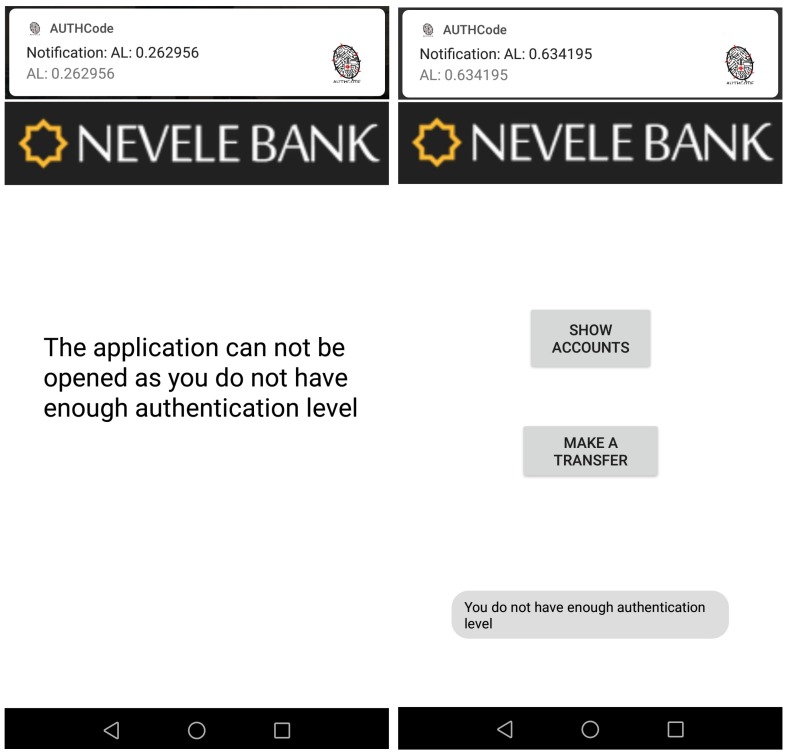
UserB using the online banking application.

A new person, UserB, uses the mobile device in his/her own way and our continuous authentication system compares the current behaviour with the well-known in real time. Since the obtained AL score is 0.26, lower than the minimum value required to open the banking application, UserB is not able to do it. The left side of Figure 12 is a screen-shot of our banking application of the Nevele bank. The upper part of the screen-shot shows a notification with the AL score obtained by our authentication system, and the lower part depicts the output of the application, indicating that it cannot be opened.

### 7.2. Sensitive Data and Transactions Are Blocked

After that, UserB simulates the UserA’s behaviour by using the same applications. However, since our solution considers data from sensors as well as statistical data from the applications usage, it returns an AL result of 0.63. This value is lower than 0.67, which means that UserB can open the banking application, but he/she cannot see sensitive information such as the account status. The right side of Figure 12 shows the output of our banking application in the previous case.

### 7.3. Sensitive Data and Transactions Lower than €20 Are Allowed

**Figure 13:**
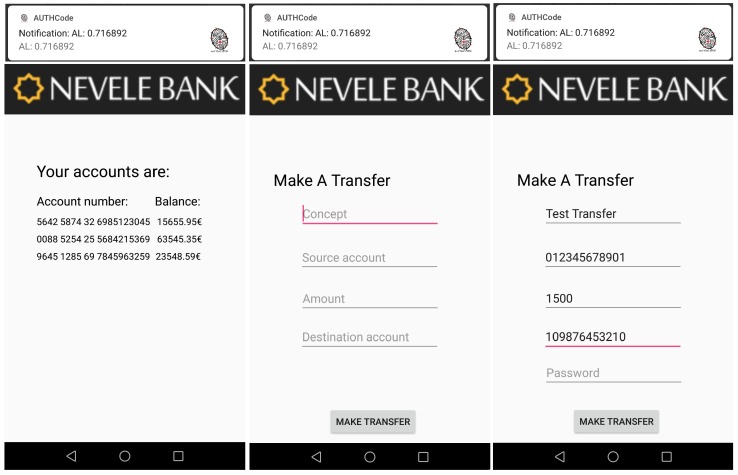
UserA using the online banking application after UserB.

In this step, the UserA starts using the mobile device in a normal way but also using a new application that has not been used during the training phase. For this point, the AL score returned by our solution is 0.71 (third threshold in Table 12). It means that UserA can see sensitive data and perform transaction lower than €20 without additional credentials. Transactions of more than this amount require an additional authentication process. The left side of Figure 13 shows when UserA accesses the account bank information, and the right one the moment when additional credentials are requested to make a transference higher than €20.

### 7.4. Full Access

**Figure 14:**
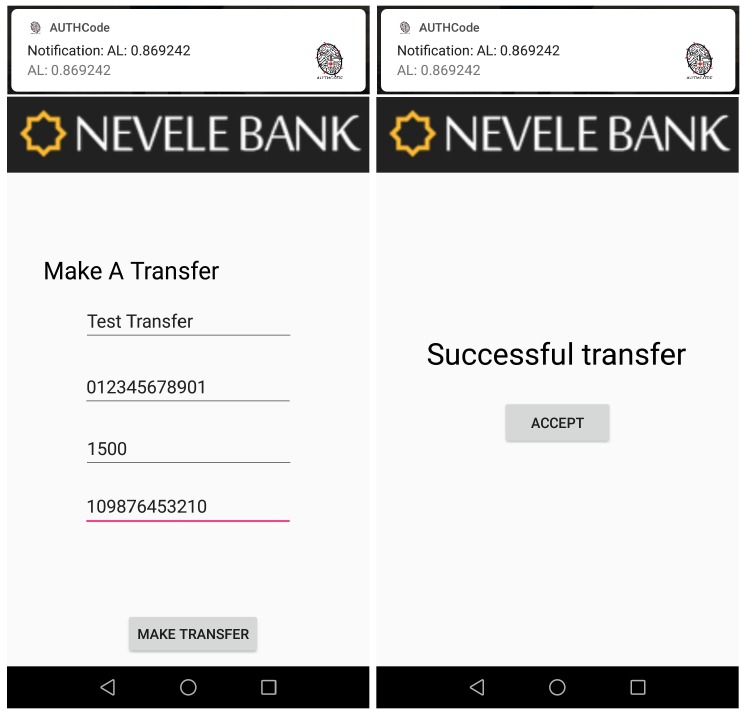
UserA using the online banking application with full access.

Finally, when UserA makes a normal use of the mobile device (as in the training process), the value obtained is higher than 0.78. It means that UserA has the possibility to access his/her sensitive information as well as making transactions of any amount without requiring additional authentication processes. Figure 14 shows when the amount of €1500 is transferred without requiring additional authentication factors or processes, since the continuous authentication system scores the current user (owner’s device) with a high authentication level.

This use case has shown the utility of our continuous authentication in a real environment, such as online banking. Specifically, we have demonstrated that it is possible to improve the user experience and the level of security by using our adaptive and continuous authentication system.

## 8. Conclusions and Future Work

In this article, we have designed, implemented and validated an intelligent and adaptive continuous authentication system for mobile devices that models the users’ behaviours by considering data coming from applications’ usage statistics and sensors. The proposed solution is able to adapt itself to changes in the user’s behaviours and uses anomaly detection based on semi-supervised ML techniques to perform the authentication process. The viability of the proposal has been demonstrated through a pool of experiments that show promising results in terms of accuracy, adaptability, resilience to attacks, and resource consumption. Finally, a use case focused on an online banking application demonstrates the utility of our continuous authentication system.

As future work, we consider extending our dataset with additional dimensions and features, such as temporal pattern information to recognize possible daily or weekly user routines, or features in frequency domain. Additionally, we plan to include data augmentation to make our system more resilient to small input variations. Our aim is to broaden the spectrum of inputs, obtaining richer profiles which make our model more robust and, at the same time, increase the authentication accuracy. Other aspect to be managed is how to calculate the optimal duration of the first phase of acquisition of behavioural data. In addition to the previous improvements, as part of our future work, we also plan to launch or solution as a final product that meets the real requirements of the current market. To that end, we intend to implement our authentication system in other platforms such as iOS as well as to evaluate the viability and usability of our solution on a larger scale. In addition, we plan to develop an API to facilitate the integration of the system with existing mobile applications that require authentication. Finally, we consider to assess our system performance with other ML algorithms that are more resource consuming when training and test computation are performed in the cloud.

## Figures and Tables

**Table 1:** Final features selected for each category.

Dimension	Features
Sensors (Gyroscope and Accelerometer)	Mean value for X, Y, Z and Magnitude (calculated as $\sqrt[{}]{X^{2}+Y^{2}+Z^{2}}$)
	Maximum value for X, Y, Z and Magnitude
	Minimum value for X, Y, Z and Magnitude
	Variance value for X, Y, Z and Magnitude
	Peak-to-peak value (max-min) for X, Y, Z and Magnitude
Application usage statistics	Number of apps and number of different apps opened for the last day and the last minute.
	App most times used and number of times used in the last minute. The same for the last day.
	Last and next-to-last apps used.
	Application most frequently used just before the currently active application.
	Bytes sent and received during the last minute.

**Table 2:** Computational complexity of IF, OC-SVM, and LOF. The variables are explained in the text.

	Computational Complexity	
Algorithm	Training	Evaluation of One Sample
Isolation Forest [37]	O(nt·log(v))	O(t·log(v))
One-Class SVM [38]	Between $O(n^{2}d)$ and $O(n^{3}d)$	$O(n_{sv}d)$
Local Outlier Factor [36]	O(1)	O(nd)

**Table 3:** Configuration parameters to train the Isolation Forest algorithm.

Method	Frequency	Parameters	Return
	After phase 1	Dataset (15 days),	
buildClassifier()	Every positive evaluation	Number of decision trees (100),	Nothing ${}^{1}$
		Sample size (256)	

${}^{1}$ Although BuildClassifier() does not return anything, this method is responsible for generating decision trees that later will be used during the evaluation phase.

**Table 4:** Configuration parameters to evaluate the Isolation Forest algorithm.

Method	Frequency	Parameters	Return
distributionForInstance()	Each 60 s	Current features vector	Double (0.0–1.0)

**Table 5:** List of initial features.

Dimensions	Features
Sensors (Gyroscope, Accelerometer and Magnetometer)	Mean value for X, Y, Z and Magnitude (calculated as $\sqrt[{}]{X^{2}+Y^{2}+Z^{2}}$).
	Maximum value for X, Y, Z and Magnitude.
	Variance value for X, Y, Z and Magnitude.
	Minimum value for X, Y, Z and Magnitude.
	Peak-to-peak (max-min) value for X, Y, Z and Magnitude.
Application usage statistics	Number of apps and number of different apps, open since the system has information. The same for the last day and the last minute.
	Apps most frequently used, average use time, and number of times used in the last minute. The same for the last day.
	Last and next-to-last apps used, number of times and average use time.
	Application most frequently used just before the currently active application and number of uses.
	Bytes sent and received during the last minute.

**Table 6:** UserA (normal) vs. UserB (anomalous) confusion matrix.

	UserB Vectors	UserA Vectors	Precision/Recall	Score
**Predicted as anomaly**	TP: 92	FP: 27	Precision	77%
**Predicted as normal**	FN: 8	TN: 73	Recall	92%

**Table 7:** Certainty classes defined for the threshold values.

Threshold	Possible User Behaviour
[0.0,0.35)	Certainly anomalous
[0.35,0.67)	Possibly anomalous
[0.67,0.78)	Possibly normal
[0.78,1.0)	Certainly normal

**Table 8:** Battery consumption of the adaptive and continuous authentication system.

Device	Total Battery	mAh Consumed	% Battery Consumed	Execution Time	Time Unlocked
Xiaomi Redmi Note 4 Pro	4180 mAh	293 mAh	7%	2 h 5 m	10 h 9 m
Huawei P10 Lite	3000 mAh	334 mAh	11%	2 h 23 m	11 h 15 m

**Table 9:** Storage consumption of our adaptive and continuous authentication system.

Device	App Dataset Size	Vectors in App Dataset	Sensor Dataset Size	Vectors in Sensor Dataset	Device Storage
Xiaomi Redmi Note 4 Pro	780 KB	8700	4 MB	13,800	64 GB
Huawei P10 Lite	164 KB	1670	1.7 MB	7500	32 GB

**Table 10:** Time consumption of our adaptive and continuous authentication system.

Device	Processor	Application Training	Application Evaluation	Sensor Training	Sensor Evaluation
Xiaomi Redmi Note 4 Pro	Mediatek Helio X20 (10 cores at 1.4 GHz)	1.5 s	1.1 ms	3.4 s	1.4 ms
Huawei P10 Lite	ARM Cortex A53 (8 cores at 2.1 GHz)	0.9 s	0.8 ms	2.1 s	1.0 ms

**Table 11:** Comparison of the different continuous authentication systems from academia and industry.

Proposal	Dimensions	Adaptive	ML Technique	Precision
Bo et al. [8]	Writing Patterns and Sensors	No	OC-SVM SVM	Accuracy: 72.36% FAR ${}^{1}$: 24.99%
Ehatisham-ul-Haq et al. [9].	Accelerometer, Gyroscope and Magnetometer	No	k-Means Bayes Net	Accuracy: 87.34–90.78%
Fridman et al. [14]	Location, Text Written, Visited Websites and Applications Usage	No	SVM	Accuracy: 95% EER ${}^{2}$: 5%
Parreño Centeno et al. [15]	Sensors	Yes	Autoencoder	Accuracy: 97.8% EER ${}^{2}$: 2%
Li et al. [17]	Gyroscope and Accelerometer	No	OneClass-SVM	FAR ${}^{1}$: 7.65% FRR ${}^{3}$: 9.01% ERR ${}^{2}$: 8.33%
De Fuentes et al. [21]	Battery, Transmitted data Ambient light, Noise	No	k-NN, Naïve Bayes, Adaptive Hoeffding Trees	Accuracy: 81.35%
Weidong Shi et al. [24]	Movement, Voice, Location and Screen touches	No	Naïve Bayes	Accuracy: 95–97%
Veridium [26]	Sensors, Camera, Touchscreen and Multiple Biometrics Factor	Unknown	Unknown	Unknown
Aware [27]	Speech Recognition, Facial Recognition, Dynamic Key and Fingerprint Recognition	Unknown	Unknown	Unknown
Zighra [29]	Location, Sensors, Newtorks and user’s writing techniques	Yes	Zigrha Algorithm	Unknown
BehavioSec [25]	Sensors, Keystrokes and Touchscreen interaction	Yes	BehavioSec algorithms	Accuracy: 97.4–99.7%
Our solution	Sensors and Applications Usage	Yes	Isolation Forest	Precision: 77% Recall: 92% Accuracy ${}^{4}$: 82.5%

${}^{1}$ FAR: False Acceptation Rate; ${}^{2}$ EER: Equal Error Rate; ${}^{3}$ FRR: False Rejection Rate; ${}^{4}$ Calculated from Table 6.

**Table 12:** Thresholds with AL score to perform different actions of the banking application.

Threshold	Possible Actions
0.0–0.35	The banking application cannot be opened
0.35–0.67	Sensitive data and transaction are blocked
0.67–0.78	Sensitive data and transactions lower than €20 are allowed
0.78–1.0	Full access
